# Anion-polarisation-directed short-range-order in antiperovskite Li_2_FeSO†

**DOI:** 10.1039/d2ta10037a

**Published:** 2023-04-12

**Authors:** Samuel W. Coles, Viktoria Falkowski, Harry S. Geddes, Gabriel E. Pérez, Samuel G. Booth, Alexander G. Squires, Conn O'Rourke, Kit McColl, Andrew L. Goodwin, Serena A. Cussen, Simon J. Clarke, M. Saiful Islam, Benjamin J. Morgan

**Affiliations:** a Department of Chemistry, University of Bath Claverton Down BA2 7AY UK swc57@bath.ac.uk b.j.morgan@bath.ac.uk; b The Faraday Institution Quad One, Harwell Science and Innovation Campus Didcot OX11 0RA UK; c Department of Chemistry, University of Oxford, Inorganic Chemistry Laboratory Oxford OX1 3QR UK; d ISIS Neutron and Muon Source, STFC Rutherford Appleton Laboratory Didcot OX11 0QX UK; e Department of Materials Science and Engineering, University of Sheffield Sheffield S1 3JD UK; f Department of Chemistry, University College London London WC1H 0AJ UK; g Department of Materials, University of Oxford Oxford OX1 3PH UK

## Abstract

Short-range ordering in cation-disordered cathodes can have a significant effect on their electrochemical properties. Here, we characterise the cation short-range order in the antiperovskite cathode material Li_2_FeSO, using density functional theory, Monte Carlo simulations, and synchrotron X-ray pair-distribution-function data. We predict partial short-range cation-ordering, characterised by favourable OLi_4_Fe_2_ oxygen coordination with a preference for polar *cis*-OLi_4_Fe_2_ over non-polar *trans*-OLi_4_Fe_2_ configurations. This preference for polar cation configurations produces long-range disorder, in agreement with experimental data. The predicted short-range-order preference contrasts with that for a simple point-charge model, which instead predicts preferential *trans*-OLi_4_Fe_2_ oxygen coordination and corresponding long-range crystallographic order. The absence of long-range order in Li_2_FeSO can therefore be attributed to the relative stability of *cis*-OLi_4_Fe_2_ and other non-OLi_4_Fe_2_ oxygen-coordination motifs. We show that this effect is associated with the polarisation of oxide and sulfide anions in polar coordination environments, which stabilises these polar short-range cation orderings. We propose that similar anion-polarisation-directed short-range-ordering may be present in other heterocationic materials that contain cations with different formal charges. Our analysis illustrates the limitations of using simple point-charge models to predict the structure of cation-disordered materials, where other factors, such as anion polarisation, may play a critical role in directing both short- and long-range structural correlations.

## Abstract 2

Anion polarisation in heterocationic Li_2_FeSO stabilises polar anion coordination in opposition to the predictions from simple point-charge electrostatics. This behaviour gives local configurational under-constraint and causes long-range disorder.

## Graphical abstract

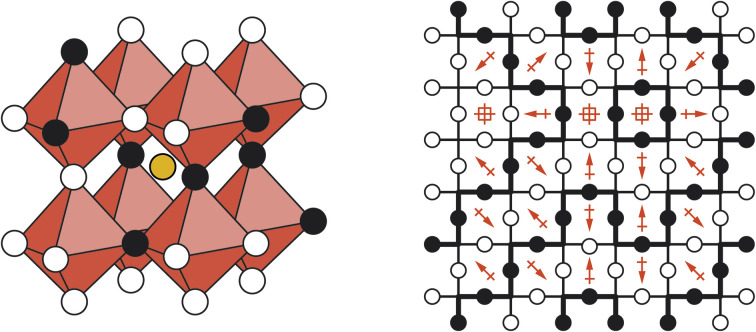

**10th anniversary statement**

We wish to congratulate the *Journal of Materials Chemistry A* on its 10 year anniversary. The continued development of novel energy materials combined and an increased understanding of their key chemical properties are critical elements in the development of a green and sustainable energy future. For the past 10 years, the *Journal of Materials Chemistry A* has provided a home for papers reporting diverse aspects of energy materials research. These papers have introduced new materials, have changed our understanding of existing classes of materials, and have steered how we think and enquire as scientific researchers. We congratulate the editorial team on their achievement, and look forward to a further 10 years of reading and contributing to the *Journal of Materials Chemistry A*.

## Introduction

Crystallographically disordered materials, in which two or more heteroatomic species are distributed over otherwise equivalent sites, find use in several applications,^1,2^ including solar cells,^3–7^ hydrogen storage,^8,9^ and lithium-ion batteries.^10–14^ The material properties of crystallographically disordered systems depend not only on their stoichiometry and crystal structure, but also on the short-range configuration of their disordered heteroatoms.^1,2,15,16^ One such example is provided by cation-disordered lithium-ion cathode materials, in which the short-range cation configuration controls lithium-transport rates, charge and discharge behaviour, and redox properties.^10,14,16–18^

A full understanding of heteroatomic materials requires both an accurate description of their short-range structures and an understanding of the physical principles that promote or inhibit specific short-range orderings. Such mechanistic understanding is particularly valuable for technologically relevant materials, where targeted synthesis protocols that promote or inhibit particular local structure motifs may allow the optimisation of key material properties.

While many anion-disordered heteroanionic materials have been structurally well-characterised,^1,2,19–22^ cation-disordered heterocationic materials have been generally less studied. For heteroanionic materials, various general design rules have been proposed to explain particular examples of partial or full anion-ordering, based on electronic, strain, or electrostatic effects.^1,2,15,20^ For heterocationic materials, however, the factors that direct short-range order preferences are less well understood.^23^

The antiperovskite-structured lithium oxychalcogenides (Li_2_M)ChO (M = a transition metal; Ch = S, Se) are one family of cation-disordered materials that have been proposed as high-capacity cathodes for lithium-ion batteries.^24–28^ The most promising of these is the oxysulfide Li_2_FeSO,^24–26^ which has a first-cycle capacity of 275 mA h g^−1^ at C/10. The practical use of Li_2_FeSO as a cathode material is limited by capacity-fade on cycling, associated with progressive amorphisation.^28^ The underlying mechanism of this cycling-induced amorphisation remains unclear; in part, because the atomic structure of pristine Li_2_FeSO is not yet fully characterised.

**Fig. 1:**
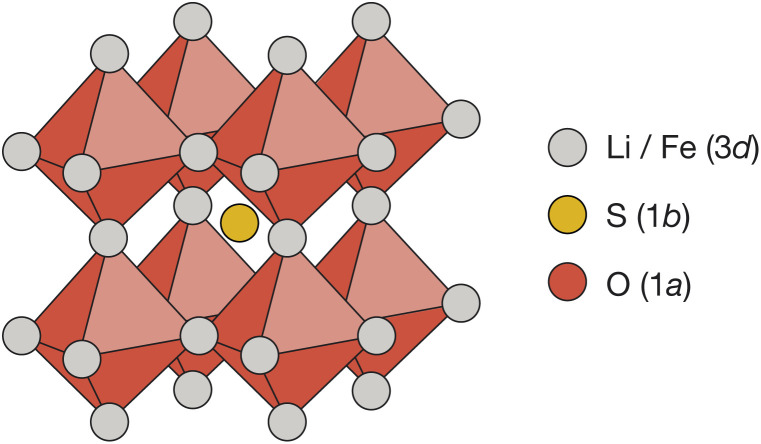
The reported antiperovskite structure (space group *Pm*3̄*m*) of Li_2_FeSO.^24^ Oxygen occupies the 6-coordinate octahedral Wyckoff 1a site. Sulfur occupies the 12-coordinate Wyckoff 1a site. Lithium and iron are distributed over the Wyckoff 3d sites in a 2 : 1 ratio.

Cubic antiperovskites, such as Li_2_FeSO, are charge-inverted structural analogues of the well-known conventional cubic perovskites. In Li_2_FeSO (space group *Pm*3̄*m*; *a* = 3.914 Å (ref. ^24^)), sulfur occupies the 12-coordinate Wyckoff 1b site, oxygen occupies the 6-coordinate Wyckoff 1a site, and lithium and iron occupy the Wyckoff 3d sites in a 2 : 1 Li : Fe ratio (Fig. 1). Previous diffraction studies of Li_2_FeSO have shown an absence of cation ordering at long ranges,^24,25^ leading to the assignment of Li and Fe as randomly distributed over the available Wyckoff 3d sites.^24^ While a “fully random” cation-distribution is consistent with the previous experimental diffraction data,^24,25^ alternative structural models with some preferential short-range order but no long-range order would also be compatible. For mixed Li/Fe systems, such as Li_2_FeSO, some degree of short-range order might in fact be expected: Fe^2+^ ions have a higher formal charge than Li^+^ ions, and simple electrostatic arguments predict that cation configurations that maximise Fe–Fe separations should be energetically favoured, resulting in some form of short-range, and possibly also long-range, cation ordering.

While previous experimental data have been interpreted as evidence of random Li/Fe distribution in Li_2_FeSO,^24^ computational studies of Li_2_FeSO have predicted that different Li/Fe configurations give different structural energies,^26,29^ indicating a preference for some specific cation configurations. This apparent inconsistency raises the question of whether the Li/Fe cation distribution in Li_2_FeSO does in fact demonstrate preferential short-range order, and, if so, what form this takes. Secondly, if Li_2_FeSO does indeed exhibit short-range cation order, what is the physical origin of this cationic ordering, and can this be explained by, for example, simple models of point-charge electrostatics?^26^

To characterise the short-range order in Li_2_FeSO, we have performed density functional theory (DFT) calculations, cluster-expansion-based Monte Carlo sampling, and X-ray total scattering experiments and pair-distribution function (PDF) analysis. Our computational model predicts partial short-range ordering, characterised by a strong preference for OLi_4_Fe_2_ oxygen-coordination, with a weaker preference for *cis*-OLi_4_Fe_2_ over *trans*-OLi_4_Fe_2_ oxygen-coordination. To validate this structural model, we have compared simulated pair-distribution functions (PDFs) against our experimental PDF data. Our DFT-derived computational model gives better agreement with the experimental data than models generated assuming either a random Li/Fe distribution or using the ground-state structure from a simple point-charge electrostatic model.

We find that Li/Fe configurations that give polar anion-coordination are stabilised relative to configurations that give non-polar-anion coordination, when compared to the energy ranking predicted by a simple point-charge electrostatic model. The stabilisation of polar Li/Fe configurations can be understood as a consequence of anions with polar coordination being electronically polarised, which lowers the net electrostatic energy for these configurations. We attribute this anion-polarisation–induced cationic short-range order in Li_2_FeSO as a consequence of high anion polarisabilities combined with a capacity for highly polar local cation configurations, and we expect this effect to be generally applicable in cation-disordered materials where the cations have different formal charges.

Finally, we discuss the role of preferential short-range order on the presence or absence of long-range order, and characterise this in terms of the configurational density of states and the temperature dependence of short- and long-range order parameters. Preferential *cis*-OLi_4_Fe_2_ oxygen coordination, as predicted by our DFT calculations, means that neighbouring pairs of OLi_4_Fe_2_ octahedra are configurationally underconstrained, and can adopt various different relative orientations. This produces long-range disorder, in agreement with experimental diffraction data, and is associated with a continuous density of states and short- and long-range order parameters that vary continuously at any non-zero temperature. In contrast, preferential *trans*-OLi_4_Fe_2_ oxygen coordination fully constrains the relative orientations of neighbouring OLi_4_Fe_2_ pairs in two dimensions, producing long-range order. In this case, short- and long-range order parameters show strong ordering to relatively high temperatures before undergoing a more sudden change to partial disorder, characteristic of a formal order–disorder transition.

Beyond the specific case of Li_2_FeSO, our results demonstrate how going beyond simple point-charge models can be necessary to understand local structure in such cation-disordered materials, and highlight the role of anion polarisation in directing short-range order, and consequently the presence or absence of long-range order, in these materials.

## Results and discussion

### Quantifying short-range order in Li_2_FeSO

The degree to which Li_2_FeSO exhibits cationic short-range order can be quantified by considering the relative probabilities of different competing cation configurations. Here, we consider the set of [Li_*x*_Fe_6−*x*_] oxygen coordination environments as a descriptor of possible short-range ordering. To sample the different local configurations expected in as-synthesised Li_2_FeSO, we first parametrise a cluster-expansion (CE) model from a set of DFT calculations with different {Li,Fe} configurations (see the Computational methods section for details), and then use the resulting DFT-CE model to perform a Monte Carlo simulation in a 8 × 8 × 8 supercell. The relative probabilities of competing OLi_*x*_Fe_6−*x*_ coordination environments are obtained directly from analysis of the Monte Carlo simulation trajectory.

**Fig. 2:**
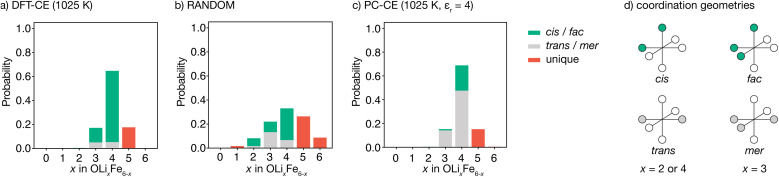
(a–c) Oxygen coordination environment probability distributions for Li_2_FeSO for (a) a density-functional theory derived cluster expansion model (DFT-CE), from Monte Carlo simulations at 1025 K, (b) random Li/Fe positions (RANDOM), and (c) an on-site point-charge cluster expansion model (PC-CE), from Monte Carlo simulations at 1025 K. (d) Schematic of *cis versus trans* (top) and *fac versus mer* (bottom) pseudo-octahedral coordination environments.

Our DFT-CE model (Fig. 2(a)) predicts that the most likely oxygen coordination is OLi_4_Fe_2_, which accounts for 65% of the oxygen environments. Within this preferential OLi_4_Fe_2_ coordination, 81% of oxygen coordination environments are *cis*-OLi_4_Fe_2_ and 19% are *trans*-OLi_4_Fe_2_. We also predict moderate amounts of OLi_3_Fe_3_ and OLi_5_Fe_2_ oxygen-coordination, with the OLi_3_Fe_3_ environments divided 71 : 29 into *fac* and *mer* configurations.

For a fully random arrangement of Li and Fe, the corresponding oxygen-coordination probability distribution is a binomial distribution with p = 2/3 and *n* = 6 (Fig. 2(b)). This distribution is visually distinct from that obtained from our Monte Carlo simulations, demonstrating the existence of short-range order in the DFT-parametrised computational model.

The preferential OLi_4_Fe_2_ coordination predicted by Monte Carlo simulation is consistent with the predictions of simple point-charge electrostatics. Coordination environments that give “local electroneutrality” are generally expected to be favoured, as expressed by Pauling's second rule.^30^ Assuming formal oxidation states, for corner-sharing O^2−^[Li_*x*_^+^Fe_6−*x*_^2+^] octahedra local electroneutrality is achieved when *x* = 4, corresponding to the preferential OLi_4_Fe_2_ coordination predicted by our DFT-CE model.

While a simple point-charge electrostatic model is consistent with preferential OLi_4_Fe_2_ coordination, this model also predicts *trans*-OLi_4_Fe_2_ as favoured over *cis*-OLi_4_Fe_2_. Considering Fe and Li as point-charges with formal 2+ and 1+ charges, respectively, that occupy ideal Wyckoff 3d crystallographic sites, the electrostatic energy of a OLi_4_Fe_2_ octahedron is minimised when the Fe ions maximise their separation by occupying opposing octahedral vertices in a *trans* configuration. Indeed, the ground state for an on-site point-charge model is comprised of 100% *trans*-OLi_4_Fe_2_ coordination. At non-zero temperatures, entropic contributions mean some proportion of non-*trans*-OLi_4_Fe_2_ oxygen coordination is expected. Yet even at relatively high temperatures, a simple point-charge model predicts a strong preference for *trans*-OLi_4_Fe_2_ over *cis*-OLi_4_Fe_2_ oxygen coordination—for a formal-charge on-site point-charge model for Li_2_FeSO with relative permittivity *ε*_r_ = 4.78 at *T* = 1025 K (Fig. 2(c)), the predicted *trans* : *cis* OLi_4_Fe_2_ ratio is 69 : 31. Even in the high-temperature limit, where the point-charge model recovers the fully random model distribution, the ratio of OLi_4_Fe_2_*trans versus cis* environments is 25 : 75, and never reaches the ratio of 19 : 81 *trans*- to *cis*-OLi_4_Fe_2_ predicted by the DFT-CE model at 1025 K.

**Fig. 3:**
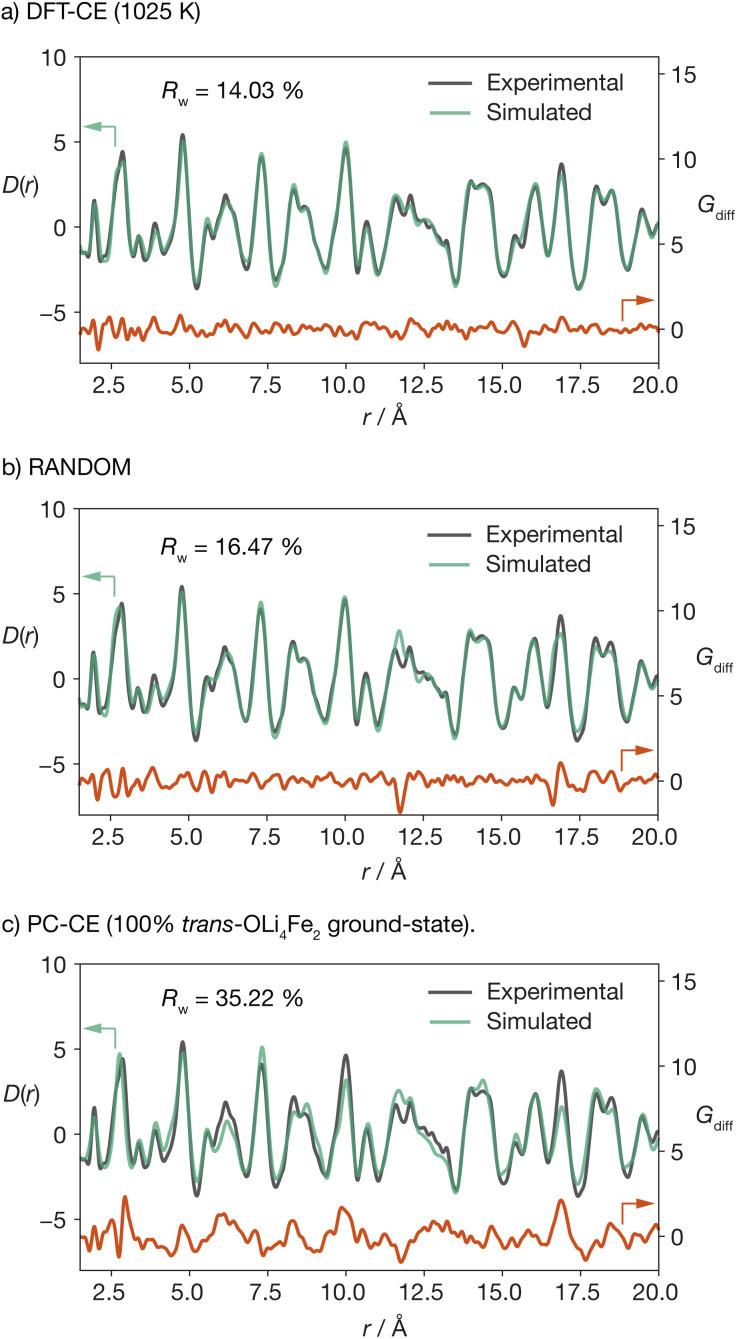
Comparison of pair-distribution functions (PDFs) obtained from X-ray total scattering and simulated PDF data for Li_2_FeSO for structural models generated for (a) the DFT-fitted cluster-expansion model (DFT-CE), (b) the RANDOM model, and (c) the PC-CE point-charge model.

To validate our DFT-CE model, we compared the pair distribution function (PDF) obtained from X-ray total scattering of Li_2_FeSO to simulated PDFs for (a) the DFT-CE model, (b) the RANDOM model, and (c) the 100% *trans*-OLi_4_Fe_2_ structure that would be obtained from a simple point-charge electrostatic ranking of all possible Li/Fe configurations (Fig. 3). The best quality-of-fit is obtained for the DFT-CE model (*R*_w_ = 14.03%), *versus R*_w_ = 16.47% for the RANDOM model, and *R*_w_ = 35.22% for the ground-state point-charge model. The particularly poor quality-of-fit for the 100% *trans*-OLi_4_Fe_2_ point-charge ground-state illustrates how a simple ranking of structures based on point-charge electrostatic energies would predict a structure that is incompatible with the experimental PDF data at short range (<20 Å).

The oxygen-coordination populations obtained from the DFT-derived cluster expansion model indicate that, on average, structures with *cis*-OLi_4_Fe_2_ are lower in energy than those with *trans*-OLi_4_Fe_2_ coordination. To better characterise the relative energies of competing OLi_4_Fe_2_ cation configurations as a function of the proportion of *cis*- *versus trans*-OLi_4_Fe_2_ oxygen coordination, we calculated the energies of all symmetry-inequivalent 2 × 2 × 2 supercells containing only Li_4_Fe_2_ oxygen-coordination—*i.e.*, every oxygen is coordinated by Li_4_Fe_2_ in either a *cis* or *trans* configuration.

**Fig. 4:**
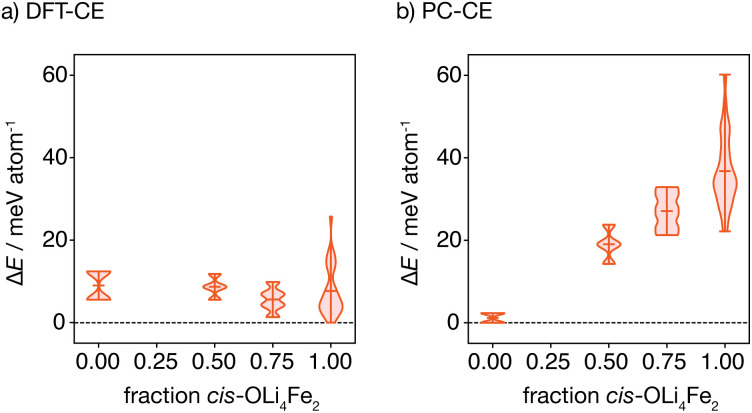
Distributions of structural energies for all 2 × 2 × 2 structures consisting solely of OLi_4_Fe_2_ oxygen coordination environments, calculated using (a) the DFT-CE DFT-fitted cluster expansion model, and (b) the PC-CE point-charge model. For each model, the distributions are subdivided according to the fraction of *cis*- *versus trans*-OLi_4_Fe_2_ octahedra in each structure.

Fig. 4(a) shows the distribution of these energies, relative to the ground-state, grouped by the proportion of *cis* oxygen coordination environments in each structure. The lowest energy structure has 100% *cis*-OLi_4_Fe_2_ coordination, as expected from the oxygen-coordination probabilities obtained from Monte Carlo simulation. Interestingly, the *highest* energy structure containing only OLi_4_Fe_2_ coordination also has 100% *cis*-oxygen coordination. This suggests the existence of a more complex relationship between cation configuration and energy than a simple preference for *cis*- *versus trans*-oxygen-coordination.

Fig. 4(b) shows the equivalent distribution of energies for all 100% OLi_4_Fe_2_ 2 × 2 × 2 supercells for the point-charge model (*ε*_r_ = 4.78). Here, the lowest energy structures are 100% *trans*-OLi_4_Fe_2_, and the energy generally increases with increasing proportion of *cis*-OLi_4_Fe_2_ coordination, as expected.

### Dipolar stabilisation of polar anion coordination

**Fig. 5:**
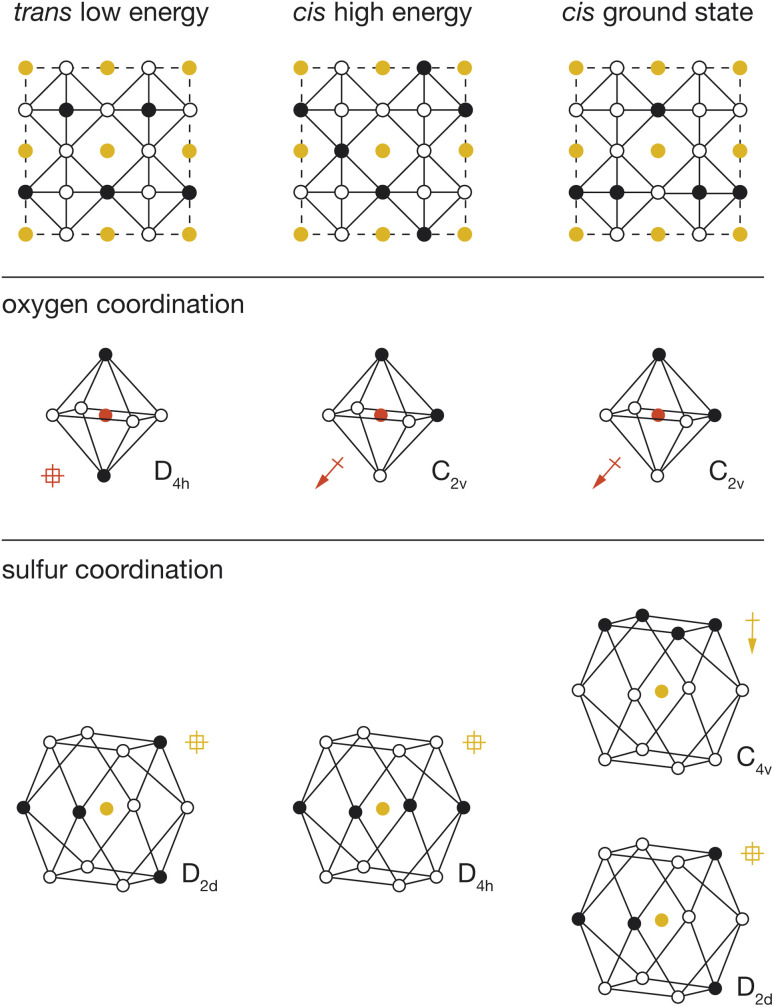
Illustration of the three structures of interest (top) with their oxygen (middle) and sulfur (bottom) environments shown with them. For the illustrated coordination environments, polar coordination environments are marked with an arrow and non-polar environments are marked with a crossed square.

To better understand the physical origin of the energy variation between structures with different OLi_4_Fe_2_ configurations, we performed further DFT calculations on three exemplar OLi_4_Fe_2_ 2 × 2 × 2 configurations: the lowest energy all-*trans* structure, the highest energy all-*cis* structure, and the lowest energy all-*cis* structure. These three structures are shown schematically in Fig. 5, with their corresponding cation configurations around oxygen and sulfur.

In our analysis, we consider two possible contributions to the total energy that are not accounted for in a simple point-charge electrostatic model. First, anions with polar coordination can move off their formal positions to give some shorter anion–cation distances, potentially resulting in stronger anion–cation interactions. Second, anions with polar coordination will be electronically polarised, and these anion dipoles will lower the net electrostatic energy of the system relative to a simple sum over point-charge Coulomb terms. This polarisation-stabilisation effect is potentially more significant in heterocationic materials, such as Li_2_FeSO, where the central atom is a relatively more polarisable anion, than in conventional heteroanionic materials, such as NbO_2_F^31^ and SrNbO_2_N,^32^ where the central atom is typically a relatively unpolarisable high-formal-charge cation.

For each DFT-optimised structure, we consider the Fe–O and Fe–S distances and the magnitudes of the O and S electronic dipoles (Table 1), which we have calculated from Wannier analysis of the converged DFT calculations. In the *trans* structure, oxygen and sulfur both have non-polar coordination, and corresponding dipole moments of 0.00 e Å. This structure can therefore be considered a reference system in which ionic and electronic polarisation effects are absent. We also obtain reference O–Fe and S–Fe nearest-neighbour distances of 1.97 Å and 2.74 Å respectively.

**Table 1:** Iron anion bond lengths and anion dipoles for the different types of anions in the different structures of interest

Structure	X	*r*(Fe–X)/Å	|*μ*_X_|/e Å
*Trans* low-energy	O_non-polar_	1.97	0.00
	S_non-polar_	2.74	0.00
*Cis* high-energy	O_polar_	1.96	0.41
	S_non-polar_	2.75	0.00
*Cis* ground-state	O_polar_	1.99	0.33
	S_polar_	2.68	0.36
	S_non-polar_	2.73	0.00

In the *cis* high-energy structure, the oxygen ions have polar coordination and exhibit a dipole moment of *μ*_O_ = 0.41 e Å. The sulfur ions have non-polar coordination, and a dipole moment of *μ*_S_ = 0.00 e Å. The O–Fe nearest-neighbour distance is effectively unchanged from the value for the reference *trans* structure, which suggests that the relative stability of this structure relative to the 100% *trans* structure cannot be attributed to a change in O–Fe distance, and instead is due to the polarisation of oxygen anions in this *cis*-OLi_4_Fe_2_-coordination structure.

In the *cis* low-energy structure, all the oxygen ions and half the sulfur ions have polar coordination, and the calculated dipole moments for these polar-coordinated anions are *μ*_O_ = 0.33 e Å and *μ*_S_ = 0.36 e Å, respectively. The polarisation of both oxygen and sulfur correlates with the lower energy of this 100% *cis*-OLi_4_Fe_2_ structure relative to the *cis* high-energy structure. In the *cis* high-energy structure, only the oxygen anions are polarised. In the *cis* ground-state structure, half the sulfur anions are also polarised, which provides additional dipole stabilisation, bringing the energy of this structure lower than that of the *trans* reference. For the *cis* ground-state structure, the O–Fe nearest-neighbour distance again is effectively unchanged from the reference *trans* value, while the S–Fe nearest-neighbour distance decreases to 2.68 Å. This off-site sulfur displacement potentially further stabilises this structure relative to the reference on-site point-charge model.

In heteroanionic oxyfluorides and oxynitrides, transition metals with polar six-coordinate *cis* or *fac* coordination move off-site to give shorter transition metal–oxygen distances, which stabilises these coordination geometries due to increased transition metal–oxygen covalent bonding.^2,22,32–36^ To characterise the degree of covalency in Li_2_FeSO, and whether this varies with different short-range anion–cation coordination configurations, we calculated integrated crystal orbital bond index (ICOBI)^37^ values for all adjacent Fe–O pairs in each of our exemplar structures. We obtain values of 0.22 to 0.25 for the three structures, which are indicative of Li_2_FeSO being highly ionic‡‡The ICOBI values reported here are comparable to those of ionic salts, such as LiCl.^37^ (see the ESI for details†).

The observation that anions with polar coordination are themselves electronically polarised explains the average increased stability of *cis*-OLi_4_Fe_2_ structures relative to *trans*-OLi_4_Fe_2_ structures, when comparing DFT-predicted energies to the corresponding energies from the simple-point charge model (Fig. 4). The formation of dipoles lowers the energy of a given structure relative to the corresponding point-charge model energy, which does not account for electronic polarisation. The *cis*-OLi_4_Fe_2_ structures have polar oxygen coordination and exhibit strong oxygen polarisation, while the *trans*-OLi_4_Fe_2_ structures have non-polar oxygen coordination and corresponding negligible oxygen polarisation. On average, therefore, all *cis*-OLi_4_Fe_2_ structures are more stable, relative to the *trans*-OLi_4_Fe_2_ structures, than would be expected from simple point-charge electrostatics.

In structures where oxygen and sulfur both have polar coordination, both anions are polarised, giving greater net stabilisation relative to the point-charge model. The effect of joint oxygen and sulfur polarisation is highlighted by comparing the relative energies of the *cis* ground-state and *cis* high-energy structures described above, predicted by the point-charge model *versus* the full DFT calculations. The point-charge model predicts that the *cis* ground-state structure has a *higher* energy than the *cis* high-energy structure (Δ*E* = 36.0 meV per atom), while the DFT calculations predict that the *cis* ground-state structure is more stable by Δ*E* = −25.7 meV per atom; we attribute this inversion of relative energies between the point-charge model and DFT data to the additional stabilising effect of sulfur polarisation in the *cis* ground-state structure.

### Short-range ordering dictates long-range ordering

The results presented above predict that Li_2_FeSO exhibits short-range order characterised by a dual preference for OLi_4_Fe_2_ oxygen coordination and for polar cation coordination around the O and S anions, *e.g.*, *cis*-OLi_4_Fe_2_ over *trans*-OLi_4_Fe_2_. The preference for polar cation-coordination around the anions is attributed to the capacity for these asymmetrically-coordinated anions to then polarise, with the resulting dipoles lowering the net electrostatic energy for these cation configurations.

**Fig. 6:**
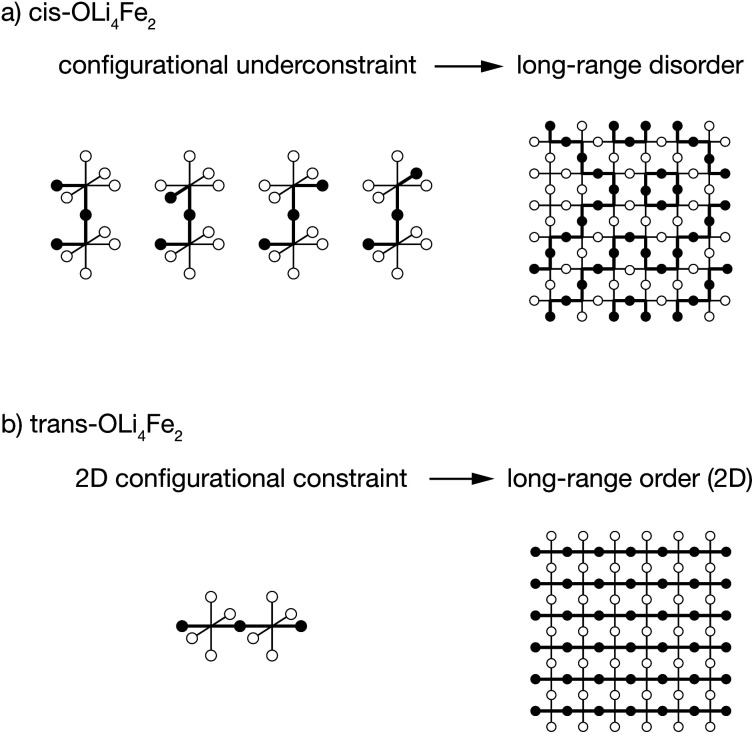
Schematic illustrating how (a) *cis versus* (b) *trans*-OLi_4_Fe_2_ short-range order produces long-range disorder—in the case of an all-*cis* ground-state—or long-range order—in the case of an all-*trans* ground state—in the limit of *T* = 0 K.

These short-range order preferences have a direct effect on the form and degree of long-range order in Li_2_FeSO. The role of preferential short-range order in directing long-range order is illustrated schematically in Fig. 6. This figure shows examples for idealised 100% *cis versus* 100% *trans*-OLi_4_Fe_2_ coordination, *i.e.*, the preferred local ordering as *T* → 0 K for OLi_4_Fe_2_ as modelled using DFT and as predicted from a simple point-charge model, respectively.

For a Li/Fe configuration with 100% *cis*-OLi_4_Fe_2_ oxygen coordination, the relative cation configurations around pairs of corner-sharing OLi_4_Fe_2_ octahedra are only partially correlated. For any individual *cis*-OLi_4_Fe_2_ octahedron, the neighbouring octahedra can adopt any of four possible configurations that preserve the local *cis* coordination. This configurational underconstraint^38^ means that even in a 100% *cis*-OLi_4_Fe_2_ system, cation site occupations progressively decorrelate with increasing cation–cation separation. At long range the Fe and Li site occupations are fully uncorrelated (Fig. 6(a)), mirroring the lack of long-range cation order observed in diffraction experiments.^24,25^

The configurational underconstraint exhibited in a 100% *cis*-OLi_4_Fe_2_ system can be contrasted with the character of a 100% *trans*-OLi_4_Fe_2_ system. For this different short-range order preference, the relative orientation of each *trans*-OLi_4_Fe_2_ coordinated octahedron fully constrains the orientations of four “in-plane” neighbouring octahedra (Fig. 6(b)) This short-range configurational constraint enforces long-range cation order in two dimensions; adjacent 2D planes can have mutually parallel or perpendicular relative orientations.§§The lowest energy 100% *trans*-OLi_4_Fe_2_ configuration for both the DFT-CE and PC-CE models corresponds to perpendicular Fe–O–Fe chains in each alternating 2D plane, which maximises the distance between Fe–Fe pairs in these adjacent layers. Hence, a structure comprised of corner-sharing A_2_B_4_-octahedra with a short-range preference for *trans*-coordination is expected to be long-range ordered, while an otherwise equivalent structure with a short-range preference for *cis*-coordination is expected to be long-range disordered.¶¶This effect, where the geometry of structural building blocks dictates the degree and form of long-range order in different materials, has previously been discussed by Overy *et al.* in the context of generalised ice rules.^39^ The all-*cis* and all-*trans* structures discussed here (Fig. 6) correspond to the C_4_C and C_4_T procrystalline systems, respectively.

**Fig. 7:**
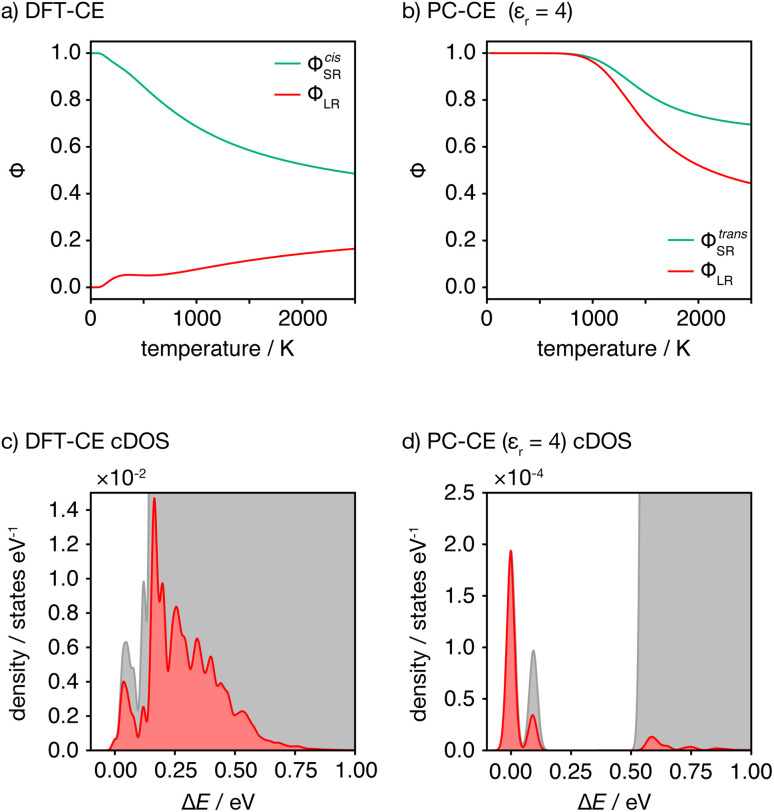
(a and b) Short- and long-range order parameters, *Φ*_SR_ and *Φ*_LR_ computed as a thermally weighted statistical average over all 2 × 2 × 2 Li_2_FeSO supercells for the DFT-CE and PC-CE (*ε*_r_ = 4.78) models. (c and d) Configurational densities of states (cDOS) for all 2 × 2 × 2 Li_2_FeSO supercells for the DFT-CE and PC-CE (*ε*_r_ = 4.78) models. Red shading indicates the distribution of thermally accessible states at *T* = 1025 K, computed by Boltzmann-weighting the full cDOS.

This analysis of idealised 100% *cis*- or *trans*-OLi_4_Fe_2_ systems illustrates that in the limit of *T* = 0 K, different short-range order preferences are predicted to lead to qualitative differences in the degree of long-range order. In as-synthesised Li_2_FeSO we expect a range of local coordination environments, as sampled in our Monte Carlo simulations. The relationship between short-range order and long-range order as a function of configurational temperature can be quantified by considering appropriate short-range and long-range order parameters. Fig. 7(a) and (b) show calculated short-range and long-range order parameters, *Φ*_SR_ and *Φ*_LR_, for the DFT-derived DFT-CE cluster expansion model and the on-site point-charge PC-CE model, respectively, calculated as a thermal average over all possible 2 × 2 × 2 Li_2_FeSO supercells. For both models the long-range order parameter is defined as the proportion of collinear Fe–O–Fe units; in a 2 × 2 × 2 supercell this is equivalent to long-range Fe–O–Fe–O–Fe ordering. The short-range order parameter in each case is defined as the proportion of *cis* or *trans* OLi_4_Fe_2_ units in the DFT-CE and PC-CE models, respectively, *i.e.*, it is the proportion of oxygen coordination environments that adopt the preferential *T* = 0 K short-range ordering.

For the DFT-CE model, at *T* = 0 K, *Φ*_SR_ = 1 and *Φ*_LR_ = 0, as expected from the discussion above (Fig. 6). As the temperature is increased, the short-range order parameter starts to decrease, even at relatively low temperatures, and the long-range order parameter shows a small increase. These short-range and long-range order parameters continuously decrease and increase, respectively, as the temperature is increased. Even at relatively high temperature, however, the long-range order parameter *Φ*_LR_ is still low, and there is no temperature regime where significant long-range ordering is predicted. In contrast, for the PC-CE (*ε*_r_ = 4.78) model, there is strong ordering at both short- and long-ranges until nearly *T* = 1000 K. Above *T* ≈ 1000 K both order parameters start to decrease. The correlation between *Φ*_SR_ and *Φ*_LR_ is expected because of the similarity in how these are defined for this system, and because of the long-range ordering promoted by preferential *trans*-OLi_4_Fe_2_ coordination.

The DFT-CE and PC-CE models therefore show somewhat qualitatively different changes in their short-range order, and hence in their long-range order, as a function of temperature. For the DFT-CE model, any increase in temperature above *T* = 0 K progressively disrupts the preferential *cis*-OLi_4_Fe_2_ coordination. For the PC-CE model, however, the short- and long-range order are resistant to thermal disordering up to *T* = 1000 K. This difference in behaviour can be understood by comparing the configurational densities of states (cDOS) for these two models (Fig. 7(c) and (d)). The DFT-CE model gives a relatively narrow cDOS. Not all cation configurations are thermally accessible at 1025 K, but the distribution of thermally accessible states forms a continuous distribution that includes a relatively large number of inequivalent cation configurations. For the point-charge PC-CE model, in contrast, the thermally accessible states at 1025 K are largely restricted to a very narrow distribution at low energy that is split off from the other states by a large energy-gap. This low energy peak consists entirely of 100% *trans*-OLi_4_Fe_2_ configurations, and we therefore observe strong long-range ordering even at 1025 K, with the sudden onset of disordering above this temperature indicative of a formal order–disorder transition.

## Summary and discussion

Here, we have reported a combined computational and experimental study of cation order in the cation-disordered heterocationic lithium-ion cathode material Li_2_FeSO. Our computational model predicts that lithium and iron cations in as-synthesised Li_2_FeSO are not randomly distributed, as previously proposed based on experimental diffraction data,^24^ but instead exhibit partial short-ranged order, characterised by a strong preference for OLi_4_Fe_2_ oxygen coordination environments, with a weaker preference for these to be *cis*-OLi_4_Fe_2_ over *trans*-OLi_4_Fe_2_. This result is supported by experimental X-ray pair-distribution function (PDF) analysis: we show that our computational model gives better agreement with these experimental PDF data than either a fully-random cation distribution, or an ordered structure constructed on the basis of point-charge electrostatics.

To better understand the physical basis for these short-range order preferences, we performed an analysis of the energies of all possible 2 × 2 × 2 Li_2_FeSO supercells, using our DFT-derived cluster-expansion model, and compared these results to the corresponding energies predicted by a simple electrostatic model, wherein the Li and Fe cations are treated as point charges fixed at their formal crystallographic positions. This analysis showed that, relative to the simple point-charge model, cation configurations with polar anion coordination, *e.g.*, *cis*-OLi_4_Fe_2_, are stabilised relative to configurations with non-polar anion coordination, *e.g.*, *trans*-OLi_4_Fe_2_.

The stabilisation of polar anion coordination environments in heterocationic Li_2_FeSO is functionally similar to the stabilisation of polar cation coordination environments in heteroanionic transition-metal oxyfluorides and oxynitrides,^2^ such as NbO_2_F^31^ and SrNbO_2_N.^32^·In Li_2_FeSO, however, this stabilisation of lower-symmetry polar coordination environments appears to have a different physical origin. In transition-metal oxyfluorides and oxynitrides, polar cation coordination environments are stabilised through off-centre displacements of the central transition-metal cation, to give enhanced covalent bonding between the central cation and the coordinating heteroanions.^22,32,34,35^ Here, we identify a different mechanism, whereby *anions* with polar heterocationic coordination are electronically polarised, with the resulting dipoles lowering the net electrostatic energy of the system. We expect this mechanism to be quite general for heterovalent (cations with different formal oxidation states) heterocationic materials. This mechanism of anion-polarisation-mediated stabilisation of polar heterocationic coordination environments requires only that the central ion is polarisable, and that polar coordination configurations of the heterocations produce an electric field. This provides a possible explanation for the difference in physical mechanism responsible for short-range order in Li_2_FeSO *versus* previously studied heteroanionic transition-metal systems: the central ions in heterocationic materials are softer more-polarisable anions, while in heteroanionic materials the central ions are typically harder less-polarisable high-valence cations.

We have also examined how the precise form of preferential short-range order dictates the presence or absence of long-range order. In Li_2_FeSO, where the preferential short-range ordering gives configurationally underconstrained preferential *cis*-OLi_4_Fe_2_ coordination, there is no long-range cation order, even at *T* = 0 K. We have contrasted this with the behaviour in a system where short-range ordering is directed purely by point-charge electrostatics. In this case, the preferential short-range ordering gives configurationally constrained *trans*-OLi_4_Fe_2_ coordination, and at *T* = 0 K the system is fully long-range ordered. By comparing different models for Li_2_FeSO we show that in the configurationally underconstrained DFT-predicted system, short- and long-range order parameters vary continuously at any non-zero temperature. In contrast, in the configurationally constrained point-charge model, short- and long-range order parameters show strong ordering to relatively high temperatures, before the onset of partial disordering. This temperature dependent onset of disorder is characteristic of a formal order–disorder transition, and can be understood as a consequence of a significant energy gap between fully ordered and disordered structures.

More generally, the results and analysis presented here illustrate how it can be necessary to go beyond simple point-charge models to predict or understand local structure in cation-disordered materials, and highlight the role of anion polarisation in directing short-range order and consequently explaining the presence or absence of long-range order.

## Methods

### Computational methods

All density functional theory (DFT) calculations were performed using the plane-wave DFT code VASP.^40,41^ Interactions between core and valence electrons were described using the projector-augmented-wave (PAW) method,^42^ with cores of [Mg] for Fe, [He] for O, [Ar] for S, and all electrons treated as valence for Li. We used the GGA functional PBEsol with a Dudarev+*U* correction applied to the Fe d states (GGA+*U*), with *U*_Fe,d_ = 5.3 eV.^43^ All calculations used a plane-wave basis-set cut-off of 720 eV. Reciprocal space was sampled using a minimum *k*-point spacing of 0.25 Å^−1^. For each structure, the ionic positions and the cell parameters were relaxed until all atomic forces were less than 1 × 10^−2^ eV Å^−1^. All calculations were spin-polarised and were initialised in ferromagnetic configurations, and then allowed to relax without electronic constraint. All calculations remained ferromagnetic during the DFT geometry optimisation.

To quantify anion polarisation in select structures we performed additional post-processing to compute the set of maximally-localised Wannier functions^44^ using the Wannier90 code.^45^ Dipoles on the ions of the cathode material are obtained by associating Wannier centres with ions and calculation of the dipole from the vectors between positively charged ionic cores and the negatively charged ionic centres. Full details are provided in the supporting dataset.^56^

To allow the computationally efficient evaluation of relative energies of Li_2_FeSO structures with different Li/Fe configurations, we parametrised a cluster-expansion effective Hamiltonian^46,47^ by fitting to the DFT-calculated energies for 111 Li_2_FeSO configurations, using the ICET and TRAINSTATION packages,^48^ with a limit of a maximum of 40 non-zero features. We use the Least Absolute Shrinkage and Selection Operator (LASSO), in combination with recursive feature elimination. The resulting cluster-expansion model gives a cross-validation score of 8 meV per atom. To construct our simple point-charge model, we fit a second cluster expansion Hamiltonian (PC-CE) to the energies obtained from an Ewald sum with a relative permittivity of *ε*_r_ = 4.78,||||*ε*_r_ = 4.78 is the electronic contribution to the static dielectric constant, calculated using dielectric perturbation theory^49^ using the HSE06 hybrid functional and a 4 × 4 × 4 *k*-point grid. for ions with formal charges positioned at their corresponding crystallographic sites. This model was fitted against all symmetry inequivalent arrangements of ions within 2 × 1 × 1 and 2 × 2 × 1 supercells.^50^ Both cluster expansions were fit using the standard sinusoidal basis function used by ICET, and considered all possible two, three, and four-body terms within cutoffs of 15 Å, 9 Å, and 5 Å respectively. For both cluster expansion models, this fitting procedure gave no non-zero four-body terms (a full description of the fitted cluster-expansion weights is available as GitHub repository information – see Data availability).

To investigate the influence of magnetic ordering on the predictive accuracy of our cluster expansion model, we considered the three exemplar structures discussed in detail in the main manuscript and performed additional calculations imposing antiferromagnetic ordering. For all three calculations, antiferromagnetic (AFM) ordering was predicted to be more stable than ferromagnetic (FM) ordering by between 7 meV per atom and 10 meV per atom, in agreement with the equivalent analysis reported in ref. ^29^. For our cluster expansion model we are interested in relative energies of different Li/Fe configurations. The change in relative energy for the three test structures produced by using energies with AFM ordering rather then FM ordering is <2 meV per atom (further details are given in the ESI†), which is both much smaller than the cross-validation score for our CE model of 8 meV per atom, and is negligible at our Monte Carlo simulation temperature of 1050 K.

To model the probable distribution of different Li/Fe configurations, we performed lattice Monte Carlo simulations using our parametrised cluster-expansion Hamiltonian, using the mchammer software package.^48^ These Monte Carlo simulations were performed in the canonical ensemble for 8 × 8 × 8 supercells using both the DFT and electrostatic fitted Hamiltonians. Initial configurations were generated at random, and then annealed from 20  000 K to 1025 K, which corresponds to the experimental synthesis temperature, with 500 000 attempted steps (approximately 32.5 MC cycles), followed by a production run at 1025 K of 1 000 000 attempted steps (approximately 641 MC cycles). Our reference distributions for fully random Li/Fe configurations were generated from a sample of 1000 random arrangements of cations. For the modelling of PDF data we generated 4 × 4 × 4 supercells using this same procedure, that were then relaxed using DFT using the same protocol as for the initial training set. For PDF modelling of a “random” Li/Fe distribution we used a special quasi-random structure^51^ within the same 4 × 4 × 4 supercell.

The three structures used for comparison with the experimental PDF were generated as follows: the 4 × 4 × 4 DFT-CE structure was generated from lattice Monte Carlo simulations using the ICET library, following the same MC protocol as described above; the 4 × 4 × 4 RANDOM structure was generated as a special quasi-random structure using ICET; the ordered *trans*-structure is the electrostatic ground state, as obtained from a full enumeration of all 2 × 2 × 2 cells, with energies calculated by Ewald summation using PYMATGEN.

### Synthesis and X-ray total scattering methods

For experimental characterisation, samples of Li_2_FeSO were prepared from stoichiometric amounts of Li_2_O (Alfa Aesar, 99.5%), Fe (Alfa Aesar, 99.9%), and S (Alfa Aesar, 99.5%) using a slightly modified version of the previously reported method.^24^ The homogenised reactants were pressed into pellets, transferred in alumina crucibles and sealed within fused silica tubes under vacuum. Deviating from the previously reported procedure, the samples were placed directly in preheated furnaces at 750 °C. The pellets were annealed for 4 h at this temperature, with intermittent grinding, and subsequently quenched in ice water. The resulting product was manually ground to obtain a fine powder. All handling of the starting materials and products was performed under dry inert gas atmosphere in an Ar-filled glove box.

X-ray total scattering data were collected at beamline I15-1 at the Diamond Light Source with an X-ray beam of energy 76.69 keV (*λ* = 0.1617 Å) and a PerkinElmer XRD 1611 CP3 area detector. Data reduction and normalisation were performed using DAWN^52^ and GudrunX^53,54^ respectively, with *Q*_min_ = 0.5 Å^−1^ and *Q*_max_ = 28.0 Å^−1^. Pair distribution function (PDF) refinements were performed using the PDFgui software.^55^ PDF fits were performed in the range 1.5 ≤ *r* ≤ 20 Å the following parameters were refined in each case: scale factor, lattice parameters *a*, *b*, and *c*, atomic correlation factor, and isotropic displacement parameters for each element. A LiFeO_2_ disordered rocksalt side phase was identified from conventional Rietveld analysis and was included in the real-space refinements.

## Data and code availability

A dataset containing the inputs and outputs for all the DFT calculations described in this paper is available from the University of Bath Research Data Archive.^56^ All code used for analysis of our raw DFT data is available in GitHub (https://doi.org/10.5281/zenodo.7828909). This analysis uses the BSYM,^50^ POLYHEDRAL-ANALYSIS,^57^ SCIPY,^58^ MATPLOTLIB,^59^ NUMPY,^60^ PYMATGEN,^61^ and ASE^62^ packages.

## Conflicts of interest

There are no conflicts to declare.

## Supplementary Material

TA-011-D2TA10037A-s001
