# Supplementary material for: Anion-polarisation-directed short-range-order in antiperovskite Li2FeSO
Source: J Mater Chem A Mater. 2023 Apr 12;11(24):13016–26. doi: 10.1039/d2ta10037a (PMC10281337; doi:10.1039/d2ta10037a)
Supplement: TA-011-D2TA10037A-s001 [file TA-011-D2TA10037A-s001.pdf]

Electronic Supplementary Material (ESI) for Journal of Materials Chemistry A.
This journal is © The Royal Society of Chemistry 2023

Supplementary Information: Anion-polarisation–directed short-range-order in antiperovskite Li_2FeSO

Samuel W. Coles,[1,2,*] Viktoria Falkowski,[3,2] Harry S. Geddes,[3,2] Gabriel E. Pérez,[4,2] Samuel G. Booth,[5,2] Alexander G. Squires,[1,6,2] Conn O'Rourke,[1,2] Kit McColl,[1,2] Andrew L. Goodwin,[3,2] Serena A. Cussen,[5,2] Simon J. Clarke,[3,2] M. Saiful Islam,[1,7,2] and Benjamin J. Morgan[1,2,†]

[1] *Department of Chemistry, University of Bath, Claverton Down, BA2 7AY, United Kingdom*
[2] *The Faraday Institution, Quad One, Harwell Science and Innovation Campus, Didcot, OX11 0RA, United Kingdom*
[3] *Department of Chemistry, University of Oxford, Inorganic Chemistry Laboratory, Oxford OX1 3QR, United Kingdom*
[4] *ISIS Neutron and Muon Source, STFC Rutherford Appleton Laboratory, Didcot OX11 0QX, United Kingdom*
[5] *Department of Materials Science and Engineering, University of Sheffield, Sheffield S1 3JD, United Kingdom*
[6] *Department of Chemistry, University College London, London WC1H 0AJ, United Kingdom*
[7] *Department of Materials, University of Oxford, Oxford, OX1 3PH, United Kingdom*

(Dated: March 25, 2023)

INTEGRATED CRYSTAL ORBITAL BOND INDEX (ICOBI) ANALYSIS

In the main manuscript we report a preference for *cis*-OLi_2Fe_4 over *trans*-OLi_2Fe_4 oxygen coordination in Li_2FeSO. Many heteroanionic materials exhibit a similar preference for *cis* coordination of anions, which is often attributed to *cis* coordination giving a stronger covalent interaction between anions and transition metal ions, due π-bonding between anion p and transition-metal d orbitals [1–6]. Here, we characterise the degree of "covalency" in Li_2FeSO by calculating integrated crystal orbital bond index (ICOBI) values [7] for the three exemplar structures described in the main manuscript (Table. I). Smaller ICOBI values indicate more "ionic" bonding. The absolute ICOBI values are similar to those calculated for LiCl, indicating that Li_2FeSO is highly ionic [7]. By comparison, the Ti–O bonds in $BaTiO_3$ have an ICOBI value 3 times higher than we obtain for Li_2FeSO [7]. This high ionic bond-character is consistent with our proposal that the preferential *cis*-OLi_4Fe_2 short-range ordering in Li_2FeSO is directed by electrostatics—specifically, anion-polarisation of anions with polar coordination environments, with a resulting electrostatic stabilisation of these coordination motifs.

Structure	r(Fe–X) / Å	ICOBI (Fe–O)
trans low-energy	1.97	0.23
cis high-energy	1.96	0.25
cis ground-state	1.99	0.22

TABLE I. Iron–oxygen bond lengths and ICOBI values for the different structures of interest.

PROJECTED DENSITIES OF STATES

Fig. 1 shows projected densities of electronic states (pDOS) for the three exemplar Li_2FeSO structures analysed in the main manuscript.

ENERGETIC DIFFERENCES DUE TO MAGNETIC ORDERING

Table. II lists the energy differences between ferromagnetic (FM) and antiferromagnetic (AFM) spin configurations for the three exemplar structures discussed in the main manuscript.

Structure	$\Delta E_{\mathrm{FM}\rightarrow\mathrm{AFM}}$ / meV atom^{-1}
trans low-energy	-7.2
cis high-energy	-10.0
cis ground-state	-8.4

TABLE II. Stabilisation energy for AFM versus FM spin ordering, $\Delta E_{\mathrm{FM}\rightarrow\mathrm{AFM}}$, for the three exemplar Li_2FeSO structures analysed in the main text.

* swc57@bath.ac.uk
† b.j.morgan@bath.ac.uk

[1] R. L. Withers, F. Brink, Y. Liu, and L. Noren, Cluster chemistry in the solid state: Structured diffuse scattering, oxide/fluoride ordering and polar behaviour in transition metal oxyfluorides, Polyhedron **26**, 290 (2007).
[2] M. Yang, J. Oró-Solé, J. A. Rodgers, A. B. Jorge, A. Fuertes, and J. P. Attfield, Anion order in perovskite oxynitrides, Nature Chem. **3**, 47 (2010).
[3] J. P. Attfield, Principles and applications of anion order in solid oxynitrides, Crys. Grow. Des. **13**, 4623 (2013).
[4] J. K. Harada, N. Charles, K. R. Poeppelmeier, and J. M. Rondinelli, Heteroanionic materials by design: Progress

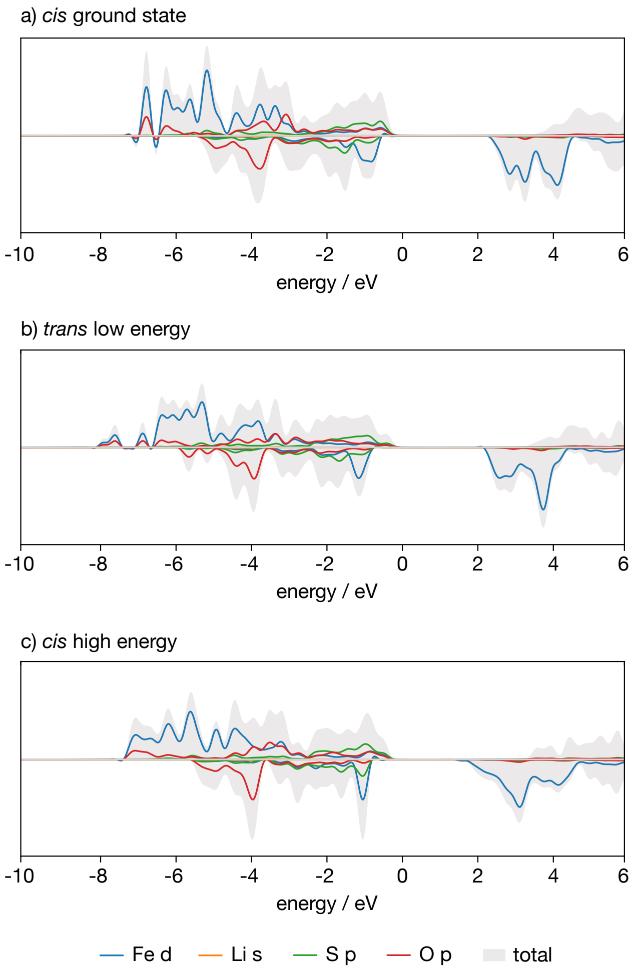

FIG. 1. Projected densities of states (pDOS) of the *cis* ground state structure (top panel), *cis* high-energy structure (middle panel), and *trans* low-energy structure (bottom panel).

toward targeted properties, Adv. Mater. **31**, 1805295 (2019).

[5] H. Wolff and R. Dronskowski, First-principles and molecular-dynamics study of structure and bonding in perovskite-type oxynitrides ABO_2N (A = Ca, Sr, Ba; B = Ta, Nb), J. Comput. Chem. **29**, 2260 (2008).

[6] C. Legein, B. J. Morgan, M. Body, W. Li, M. Burbano, M. Salanne, T. Charpentier, O. J. Borkiewicz, and D. Dambournet, Correlated anion-disorder in heteroanionic cubic $TiOF_2$ (2022), in preparation.

[7] P. C. Müller, C. Ertural, J. Hempelmann, and R. Dronskowski, Crystal orbital bond index: Covalent bond orders in solids, J. Phys. Chem. C **125**, 7959 (2021).
